# Severe acute respiratory coronavirus virus 2 (SARS-CoV-2) infection among hospital workers in a severely affected institution in Madrid, Spain: A surveillance cross-sectional study

**DOI:** 10.1017/ice.2020.1303

**Published:** 2020-10-29

**Authors:** Felipe Pérez-García, Aurora Pérez-Zapata, Naroa Arcos, Manuel De la Mata, María Ortiz, Encarnación Simón, Irene Hervás Fernández, Victoria González Ventosa, Mario Muñoz Monte, Javier González Arroyo, Ramón Pérez-Tanoira, Juan Cuadros-González

**Affiliations:** 1 Servicio de Microbiología Clínica, Hospital Universitario Príncipe de Asturias, Madrid, Spain; 2 Servicio de Prevención de Riesgos Laborales y Salud Laboral, Hospital Universitario Príncipe de Asturias, Madrid, Spain.

## Abstract

**Objective:**

To analyze the impact of the coronavirus disease 2019 (COVID-19) pandemic in workers of a hospital located in one of the most affected areas in Spain.

**Design, settings, and patients:**

Cross-sectional study performed between March and May 2020 over all workers of a secondary hospital in Madrid, Spain.

**Methods:**

We employed polymerase chain reaction (PCR, for symptomatic individuals) and serology (for both PCR-negative symptomatic workers and asymptomatic workers) as diagnostic tests for severe acute respiratory coronavirus virus 2 (SARS-CoV-2). We analyzed the prevalence of the virus in healthcare workers (HCWs) and nonhealthcare workers (nHCWs). We also collected information about the use of personal protective equipment (PPEs) and possible contacts prior to infection.

**Results:**

In total, 2,963 workers were included: 1,092 were symptomatic, and of these, 539 were positive by PCR (49.4% of symptomatic workers). From the remaining symptomatic workers, 197 (35.6%) were positive by serology. Regarding asymptomatic workers, 345 were positive by serology (31.9% of infected workers). In total, 1,081 (36.5%) presented a positive diagnostic test for SARS-CoV-2. Infection rates were different between HCWs (37.4%) and nHCWs (29.8%) (*P* = .006). In the multivariate logistic regression analysis, the use of PPE (protective: OR, 0.56; 95% CI, 0.44–0.72; *P* < .001) and previous contact with COVID-19 patients (risk factor: OR, 1.69; 95% CI, 1.28–2.24; *P* < .001) were independent factors that were associated with SAS-CoV-2 infection.

**Conclusions:**

Overall, >36% of our workers became infected with SARS-CoV-2, and the rate of asymptomatic infections accounted for almost 32% of all SARS-CoV-2 infections. We detected differences in the rates of infection between HCWs and nHCWs. The use of PPE and previous contact with COVID-19 patients were associated with SARS-CoV-2 infection.

The impact of the COVID-19 pandemic on healthcare workers (HCWs) has been immense according to data from China,^1–3^ Europe,^2^ and the United States, where the CDC reported 91,267 COVID-19 cases and 501 deaths as of June 23, 2020.^4^ In Spain, as of June 25, 2020, ~52,500 cases in HCWs have been reported, accounting for >20% of the total cases in the general population.^5^

Different factors facilitated the rapid spread of the infection in hospital settings, such as the transient unavailability of personal protective equipment (PPE)^6^ and the initial assumption that the main transmission route of the virus was only by droplets.^7^ These facts delayed the generalized used of surgical masks inside hospitals.^8^ Northeast of Madrid has been one of the most severely affected regions, with a cumulative incidence of 1,300 cases per 100,000 inhabitants in our health area (Alcalá de Henares)^9^ and a mortality rate of 27% among patients that were attended at our emergency department.^10^

The Ethics Committee of Hospital Universitario Príncipe de Asturias (Madrid) approved the study (protocol number: no. COVID-HUPA).

In this study, we aimed (1) to assess the prevalence of infection among hospital workers, (2) to describe the different groups of infected workers, and (3) to assess the risk factors that were associated with SARS-CoV-2 infection in our setting.

## Material and methods

### Population and study period

We performed a study from March 5, 2020, to May 30, 2020, among workers from our hospital: 490 beds and almost 3,100 hired workers. To evaluate the impact of SARS-CoV-2 pandemic in our hospital, we recorded the number of daily cases by polymerase chain reaction (PCR), differentiating those belonging to hospital workers. The study was performed in 2 periods. During the first period (March and April), PCR was performed in nasopharyngeal exudate for all hospital workers who presented symptoms of SARS-CoV-2 infection and were attended by the occupational health department. During the second period (May), all remaining workers were studied by serology (IgM and IgG antibodies), including asymptomatic workers and symptomatic workers that were negative by PCR.

All workers with a previously confirmed diagnosis of COVID-19 by PCR were not tested using serology. The serologic test was performed using finger-prick blood, and results were immediately reported by the Occupational Health practitioners. Workers were informed about the results and its interpretation. All workers with positive IgM antibodies were additionally studied using PCR. A case of SARS-CoV-2 infection was defined for those workers who presented a positive PCR or serologic test.

### Clinical and occupational data

Demographic and clinical data, professional category, and epidemiological data were extracted from the database of the Occupational Health Service. Demographic and clinical characteristics included age, gender, comorbidities (ie, smoker, hypertension, diabetes, cardiovascular disease, chronic obstructive pulmonary disease–COPD, pregnancy and immunosuppression) and clinical features for symptomatic workers (ie, symptoms and disease severity). Hospital workers were categorized as HCWs (including medical staff, nursing, technical specialist, auxiliary nursing care technician and hospital porters) and nHCWs (including kitchen personnel and administrative staff).

Epidemiological data included reported use of PPE, the participation in aerosol generating procedures (AGP) and previous risk contacts with COVID-19 patients, coworkers, or relatives, and type of contact (close or casual contact, according to standardized protocols).^11^ Briefly, a close contact was defined for any individual who had contact with a case from 48 hours before the onset of symptoms until case isolation (ie, providing care to the case without adequate protection measures or being in the same place that a confirmed case, such as cohabiting or visits, at a distance of <2 m for >15 minutes). On the other hand, casual contact was defined for those individuals that had been in the same closed space with a symptomatic case, without meeting criteria for close contact.^11^

### Diagnostic methods

#### Molecular techniques

Diagnosis of SARS-CoV-2 infection by PCR was based on the detection of the E, N, ORF1ab, or RdRP genes. RNA amplification was made using 2 real-time PCR platforms: VIASURE SARS-CoV-2 Real-Time PCR Detection Kit (Certest Biotech, Zaragoza, Spain) and Allplex 2019-nCoV assay (Seegene, Seoul, South Korea). All equipment was used according to the manufacturer’s instructions for both the handling and the interpretation of the results.

#### Serology

For the seroprevalence study, we used the AllTest COVID-19 IgG/IgM kit (AllTest Biotech, Hangzhou, China). This test consists of a qualitative membrane-based immunoassay (immunochromatography or lateral flow immunoassay, LFA) for the detection of IgG and IgM antibodies against SARS-CoV-2 in whole blood, serum, or plasma samples. This LFA was previously validated in our hospital showing a specificity of 100% and a sensitivity of 88% from 14 days after the onset of symptoms.^12^ We used 20 µL of finger-prick whole blood obtained for these tests.

### Statistical analysis

Continuous variables were expressed as median and interquartile range (IQR) and categorical variables as proportions. For the interpretation of serology, we considered a positive result for samples in which IgG, IgM, or both were detected. Comparisons between groups were made using the Mann-Whitney U test for continuous variables and the 2-tailed Fisher exact test for categorical variables. Comparisons for demographical and clinical date were calculated using the Kruskal-Wallis test for continuous variables and the 2-tailed Fisher exact test for categorical variables using the noninfected individual’s category as a reference. *P* values were corrected for multiple comparisons using the Bonferroni procedure. For these comparisons, *P* ≤ .05 was considered significant.

Logistic regression analysis was performed to establish which variables were associated with SARS-CoV-2 infection, for the following variables: type of hospital worker (HCW vs nHCW), professional category (using medical staff as a reference), type of contact (close vs casual), use of PPE, performance of AGP, contact with COVID-19 patients, contact with hospital coworkers, and contact with relatives. A multivariate logistic regression test was adjusted by the most significant covariates, which were selected using a stepwise method (forward). Results for regression analysis were expressed as odds ratios (ORs), and 95% confidence intervals (95% CIs) were calculated for each risk factor using the Wald approximation method. Statistical analyses were performed using Stata/IC version 13.1 software (StataCorp, College Station, TX).

## Results

### Impact of SARS-CoV-2 pandemic and results of the diagnostic techniques

**Fig. 1.**
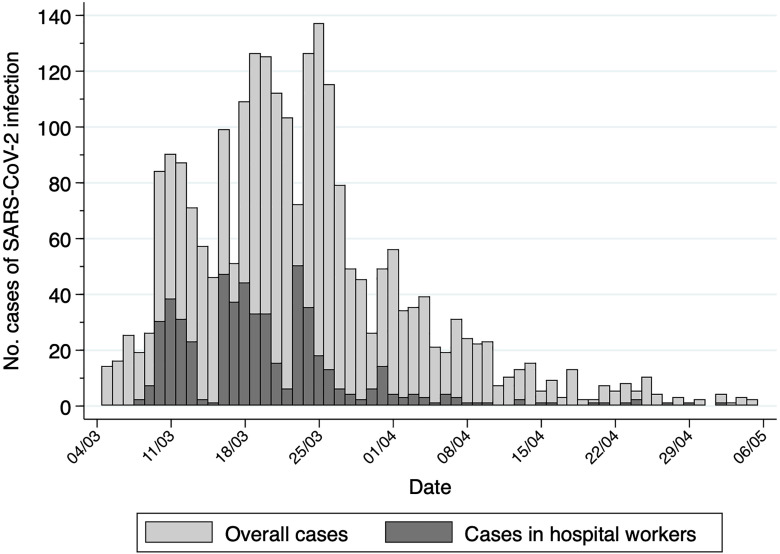
Impact of SARS-CoV-2 pandemic in our hospital. This figure shows only the cases of SARS-CoV-2 infection diagnosed by polymerase chain reaction (PCR). Each case represents a new diagnosis as duplicates in the PCR tests were eliminated for this analysis.

Figure 1 shows the impact of the pandemic from March 5, 2020, to May 5, 2020, in our hospital. During this period, the clinical microbiology department performed >6,000 PCR assays, and 2,397 cases of SARS-CoV-2 infection were diagnosed using this method. During the first 2 weeks, 1,883 new cases of SARS-CoV-2 infection were detected, and 477 (25.3%) of these were hospital workers. Management of COVID-19 patients was conducted following protocols established by our government (Supplementary Table 1 online).

**Fig. 2.**
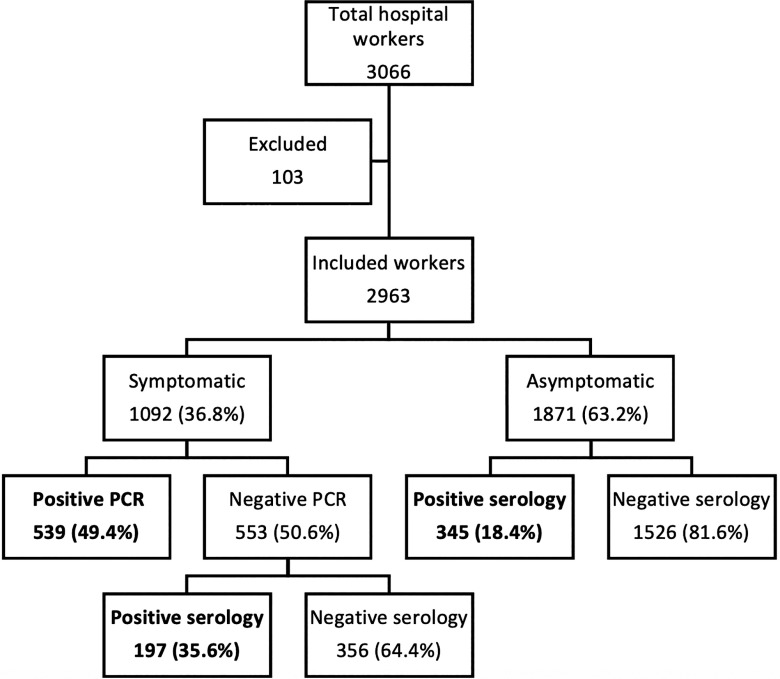
Results of the study on hospital workers. In total, 103 workers could not be included in the survey and, therefore, where excluded from the analysis: 31 workers had symptoms of COVID-19 but without serologic test performed after a negative PCR; 10 symptomatic workers had no PCR or serology test performed; 62 asymptomatic workers had no serology test.

The main results of the study are summarized in Figure 2. Briefly, our hospital had 3,066 workers at the beginning of the study. However, 103 workers could not be enrolled, so they were excluded from the analysis, for a final study population of 2,963 workers. Moreover, 1,092 (36.8%) workers presented symptoms compatible with COVID-19, of whom 539 (49.4%) were positive for SARS-CoV-2 by PCR. Among symptomatic workers with negative PCR results (553 workers, 50.6% of symptomatic population), 197 (35.6%) had a positive result in the subsequent serologic test. Regarding the remaining 1,871 asymptomatic workers, serology was positive for 345 of them (18.4% of asymptomatic workers, accounting for 31.9% of total SARS-CoV-2 infections). Regarding the results of serology, 2,424 tests were performed,and 542 (22.4%) were positive. The vast majority of positive workers (487, 89.9%) were positive only for IgG antibodies; 40 (7.4%) were positive for both IgM and IgG antibodies; and 15 (2.8%) were positive only for IgM. All 55 workers with positive IgM tests underwent a SARS-CoV-2 PCR test to exclude active infection and these results were negative for all of them. Taking into account the results of both PCR and serology, 1,081 hospital workers (36.5%) of our institution had evidence of infection by SARS-CoV-2.

### Characteristics of the different groups of infected workers

Workers without evidence of SARS-CoV-2 infection presented some differences from those who became infected (Table 1). No differences were detected in gender, pregnancy, or immunosuppression between any of the 3 groups of infected workers and the noninfected group. Compared with noninfected workers, symptomatic workers with positive PCR tests were older; they were less frequently smokers; and they more frequently presented comorbidities such as hypertension, diabetes, COPD, or immunosuppression (Table 1). Symptomatic workers with negative PCR and positive serology tests were older and more frequently presented hypertension and COPD than noninfected individuals. Finally, asymptomatic workers with positive serology tests presented fewer differences in comorbidities compared with noninfected individuals, who showed differences only in smoking habit (Table 1).

**Table 1.** Characteristics of the Different Groups of Infected Workers^a^

Characteristics	Without Evidence of Infection	With Evidence of Infection					
		Symptomatic With Positive >PCR	*P* Value	Symptomatic With Positive Serology	*P* Value	Asymptomatic With Positive Serology	*P* Value
No. workers	1,882	539	…	197	…	345	…
Sex, female	1,521 (80.8)	438 (81.3)	1.000	157 (79.7)	1.000	271 (78.6)	1.000
Age, median years (IQR)	42.6 (29.9–54.8)	45.6 (35.7–54.9)	**.003**	50.0 (36.9–57.3)	**< .001**	38.6 (28.5–52.1)	.077
**Underlying conditions**							
Smoker	284 (15.1)	54 (10.0)	**.007**	19 (9.6)	.130	25 (7.3)	**< .001**
Hypertension	97 (5.2)	57 (10.6)	**<.001**	19 (9.6)	**.041**	19 (5.5)	1.000
Diabetes	32 (1.7)	21 (3.9)	**.012**	7 (3.6)	.268	5 (1.5)	1.000
Cardiovascular disease	32 (1.7)	15 (2.8)	.338	4 (2.0)	1.000	6 (1.7)	1.000
COPD	57 (3.0)	33 (6.1)	**.005**	15 (7.6)	**.009**	8 (2.3)	1.000
Pregnancy	12 (0.6)	8 (1.5)	.188	3 (1.5)	.491	3 (0.9)	1.000
Immunosuppression	14 (0.7)	12 (2.2)	**.021**	2 (1.0)	1.000	0 (0.0)	.439

Note. *P* value, level of significance; IQR, interquartile range; COPD, chronic obstructive pulmonary disease.

a Statistics: Values are expressed as median (IQR) and absolute count (percentage). *P* values were calculated using the 2-tailed Fisher exact test for categorical variables and the Kruskal-Wallis test for continuous variables using the noninfected individual’s category as reference and were corrected for multiple comparisons using the Bonferroni procedure. Significant differences are shown in bold.

Regarding symptomatic workers, Supplementary Table 2 (online) summarizes the comparisons between those positive by PCR or serology. Clinical courses were similar for both groups, but those symptomatic workers with positive PCR results presented slightly higher percentages of fever (>38°C) and headache and notably higher percentages of anosmia and ageusia (Supplementary Table 2). Symptomatic workers with positive PCR tests had higher rates of hospital admission, but they did not have worse outcomes; pneumonia and ICU admission rates were almost the same for both subgroups. None of the workers died from COVID-19, and at the end of this study all who had been admitted to the hospital had already been discharged.

### Impact of SARS-CoV-2 infection on different workers

The Distribution of COVID-19 cases is summarized in Table 2. Among the 2,614 HCWs, 977 (37.4%) became infected. Regarding nHCWs (349 workers), 104 (29.8%) were infected by SARS-CoV-2 and the difference in percentages of infection between HCW and nHCW was statistically significant (*P* = .006). In the group of HCWs, the most affected professional categories were hospital porters (83 cases, 44.9%), followed by nurses (340 cases, 39.6%) and auxiliary nursing care technicians. Regarding nHCWs, kitchen personnel were the most affected category (26 cases, 33.3%), followed by administrative staff (59 cases, 30.3%).

**Table 2.** Infection Rates by Professional Categories

Professional Category	Workers Per Category	Infected Workers, No. (%)
Healthcare personnel (HCWs)	**2,614**	**977 (37.4)**
Medical staff	444	154 (34.7)
Nurses	859	340 (39.6)
Technical specialist	141	41 (29.1)
Auxiliary nursing-care technician	641	250 (39.0)
Hospital porter	185	83 (44.9)
Resident physician	272	90 (33.1)
Others^a^	72	19 (26.4)
Non-healthcare personnel (nHCWs)	**349**	**104 (29.8)**
Kitchen	78	26 (33.3)
Administrative staff	195	59 (30.3)
Others^b^	76	19 (25.0)
Overall	**2,963**	**1,081 (36.5)**

a This category included mainly physiotherapists, psychologists and midwives.

b This category included maintenance personnel, engineers and cleaning personnel.

### Risk factors associated with SARS-CoV-2 infection

The results of the logistic regression analysis of risk factors associated with infection are summarized in Table 3. Univariate analysis showed that type of hospital worker (ie, HCW), professional category (ie, hospital porters) and performance of AGP were significantly associated with infection, whereas the type of contact (close vs casual) was not statistically associated with infection. The use of PPE was a protective factor against infection (OR, 0.62; 95% CI, 0.48–0.079; *P* < .001). Recommended PPE for care of COVID-19 patients in the methods is summarized at the end of Table 3. Regarding previous reported contacts, contact with COVID-19 patients was more strongly associated with SARS-CoV-2 infection (OR, 5.10; 95% CI, 4.30–6.06; *P* < .001) than other reported contacts.

**Table 3.** Risk Factors Associated With SARS-CoV-2 Infection in Our Workers

Risk Factor	Univariate analysis^a^	
	OR (95% CI)	*P* Value
Type of hospital worker (HCW vs nHCW)	1.41 (1.10–1.79)	.006
Professional category (compared with medical staff)		
Nurses	1.23 (0.97–1.57)	.084
Technical specialist	0.77 (0.51–1.17)	.219
Auxiliary nursing care technician	1.20 (0.94–1.55)	.148
Hospital porter	1.53 (1.08–2.17)	**.017**
Resident physician	0.93 (0.68–1.28)	.662
Other HCWs^b^	0.68 (0.39–1.18)	.169
Kitchen	0.94 (0.57–1.57)	.817
Administrative staff	0.82 (0.57–1.17)	.275
Other nHCWs^c^	0.63 (0.36–1.09)	.100
Use of PPE^d^	0.62 (0.48–0.079)	**<.001**
Participation in AGP	2.54 (1.71–3.77)	**<.001**
Type of contact (close vs casual)	1.17 (0.087–1.57)	.309
Contact with COVID-19 patients	5.10 (4.30–6.06)	**<.001**
Contact with coworker	3.18 (2.64–3.82)	**<.001**
Contact with relatives	2.16 (1.50–3.11)	**<.001**
	Multivariate analysis	
Use of PPE	0.56 (0.44–0.72)	**<.001**
Type of contact (close vs casual)	1.32 (0.97–1.80)	.081
Contact with COVID-19 patients	1.69 (1.28–2.24)	**<.001**

Note. HCW, healthcare worker; nHCW, nonhealthcare worker; PPE, personal protective equipment; AGP, aerosol-generating procedure; *P* value, level of significance; OR, odds ratio; 95% CI, 95% confidence interval.

a Results for regression analysis are expressed as odds ratios (ORs). The 95% confidence intervals were calculated for each risk factor using the Wald approximation method. Significant differences are shown in bold. Multivariate analysis results show the most significant covariates, which were selected by a stepwise method (forward).

b This category included mainly physiotherapists, psychologists and midwives.

c This category included maintenance personnel, engineers and cleaning personnel.

d Recommended PPE for care of COVID-19 patients consisted of disposable medical caps, FFP2 masks, disposable medical protective clothing (waterproof), disposable gloves and protective goggles or protective screens. When an AGP was necessary, the involved workers wore FFP3 masks.

In the multivariate analysis, only the use of PPE (protective: OR, 0.56; 95% CI, 0.44–0.72; *P* < .001) and previous contact with COVID-19 patients (risk factor: OR, 1.69; 95% CI, 1.28–2.24; *P* < .001) were independent factors associated with SARS-CoV-2 infection, whereas type of contact (close vs casual) did not reach statistical significance (*P* = .081).

## Discussion

Our study shows that our institution has suffered a huge impact of the COVID-19 pandemic; more than one-third of our workers have been infected with SARS-CoV-2. We found statistically significant differences in the rates of infection between HCWs and nHCWs. The use of PPE and a previous contact with COVID-19 patients were independent factors that were associated with SARS-CoV-2 infection.

The disparity in infection prevalence in hospital workers across Europe is high. For instance, in a German tertiary-care hospital located in an area with little impact of the pandemic, the prevalence of infected HCWs was only 1.6%.^13^ In another study carried out in a hospital specialized in infectious diseases in Naples, the prevalence was 3.4%.^14^ Finally, another group of researchers in Brussels reported 12.6% infection at their institution.^15^ In Spain, it is estimated that ~5% of the general population has antibodies against SARS-CoV-2 and in Madrid, this percentage increases to 11%.^16^ Regarding data of infections in HCWs in Spain, 2 different studies have been published. On the one hand, the study of Folgueira et al^17^ was performed only using RT-PCR and showed that 11.6% of all hospital workers had a PCR test positive for SARS-CoV-2. On the other hand, García-Basteiro et al^18^ also reported an 11.2% infection prevalence combining PCR and serology tests. In our hospital, combining PCR in symptomatic personnel and serology screening in both symptomatic and asymptomatic workers, we reached a prevalence of SARS-CoV-2 infection of 36.5%. These results contrast with those of other Spanish studies that show a prevalence 3 times lower. However, our results are from one of the most affected communities of Spain (Alcalá de Henares, Madrid), which had a cumulative incidence of 1,300 cases per 100,000 inhabitants as of June 28, 2020.^9^ Additionally, the rate of infection in hospital workers increased in parallel to the number of infected patients (Fig. 1).

This high rate of infection could reflect also the problem associated with the lack of proper PPE at the beginning of the pandemic,^6^ the initial recommendations against the generalized use of face masks,^7,8^ and/or the probable silent dispersion of the virus in the society and the hospital long before the first cases were detected by molecular methods.

This explanation is in line with our findings; in multivariate analysis, the use of PPE was an independent protective factor for SARS-CoV-2 infection (OR, 0.56; 95% CI, 0.44–0.72; *P*< .001). This finding also reflects the need to provide adequate PPEs to hospital workers to control the spread of the pandemic in this environment.

We found a high rate of false-negative results of PCR in symptomatic workers who later developed a serological response to SARS-CoV-2 (35.6%). This difference between analytical and clinical sensitivity of PCRs is widely known and has previously been described.^19^ Another study performed at our institution showed that almost 90% of 63 clinical COVID-19 pneumonias, which tested negative by PCR for SARS-CoV-2, could be diagnosed through a rapid test detecting IgG and IgM antibodies.^12^ Our results reinforce the need to use tests complementary to PCR, such as serology, for initial diagnosis of SARS-CoV-2 infection.^20,21^

Our study also points out the great problem posed by individuals with asymptomatic infection: in our hospital up to 31.9% of infections in workers were asymptomatic. As a consequence, for future epidemic waves, screening should cover all personnel, not just symptomatic individuals because those with asymptomatic infections also spread the virus.^22^

We observed different infection rates between HCWs (37.4%) and nHCWs (29.8%). As expected, nurses, auxiliary nursing-care technicians, and medical staff presented higher rates of infection among HCWs (39.6%, 39.0%, and 34.7%, respectively), reflecting their close contact with COVID-19 patients. However, the most affected professional category in our institution were hospital porters (44.9%). There is some debate about whether hospital porters could be considered HCWs or nHCWs. However, beyond formal considerations, in our opinion, the work of hospital porters frequently involves contact with patients at short distance. This may explain why hospital porters were the most affected professional category in our hospital and indicates that they should be considered at-risk personnel who require adequate PPE. At the beginning of the pandemic in Wuhan, other researchers established that infections among HCWs occurred mainly in low-contagion areas, which supported the need for routine screening to detect asymptomatic carriers.^23^ Our results are in line with these findings—they reflect the need for screening in all professional categories in addition to that classically recognized to be at risk (nurses and physicians) as well as the importance of establishing an adequate workflow to interrupt nosocomial transmission routes.

Our study has some limitations. First, it was a retrospective analysis conducted in a single hospital. Consequently, we could not present data about the kind of PPE that was employed or the existence of possible breaches in PPE use. Further prospective multicenter studies are necessary to reinforce our findings. Second, our serologic results are based on the use of a lateral flow immunoassay (All Test COVID-19 IgG/IgM). The usefulness of this kind of test has been questioned due to a lack of official performance validations.^24^ However, recently published studies have shown that these point-of-care tests could be a suitable option for seroepidemiological studies.^16^ Furthermore, we previously performed a validation of the test using 100 pre-pandemic sera and 90 serum samples from patients with positive PCR for SARS-CoV-2, and we found a specificity of 100% and a sensitivity of 88% from 14 days after the onset of symptoms. Thus, we concluded that this serologic test was reliable to diagnose SARS-CoV-2 infection and could be used for the seroprevalence study.^12^ However, some techniques based on ELISA or chemiluminescence could present sensitivities of almost 100%,^25^ and a second seroprevalence study with these techniques in our institution could yield interesting findings.

Despite these limitations, to the best of our knowledge, our study constitutes one of the largest cohorts regarding SARS-CoV-2 infection among hospital workers. Moreover, it was carried out on the entire hospital staff and provides relevant data about symptoms, comorbidities, infected professional categories, use of PPE, as well as contact investigation in one of the institutions that have been most affected by the pandemic.

Hospital workers are the first-line workforce for clinical care of COVID-19 patients; therefore, their security at work must be ensured to warrant the success facing the SARS-CoV-2 pandemic.^26^ During the first phase of the pandemic, the availability of PPE was quite limited in most European countries, and many hospital workers were supplied with equipment that might not meet protection standards. Adequate provision of PPE and individual support in terms of rest, family, and psychological support must be ensured by the hospital management.^26,27^ The danger of a second wave is real, and hospitals should establish a preparedness plan to minimize the impact of this highly contagious disease to protect their most valuable asset—their workers.^28^

## Supplementary material

For supplementary material accompanying this paper visit https://doi.org/10.1017/ice.2020.1303. click here to view supplementary material
