# Supplementary material for: Severe acute respiratory coronavirus virus 2 (SARS-CoV-2) infection among hospital workers in a severely affected institution in Madrid, Spain: A surveillance cross-sectional study
Source: Infect Control Hosp Epidemiol. 2020 Oct 29:1–7. doi: 10.1017/ice.2020.1303 (PMC7691660; doi:10.1017/ice.2020.1303)
Supplement: Supplementary file 1 [file S0899823X20013033sup.zip › S0899823X20013033supp001.docx]

**Supplementary Table 2. Clinical features of symptomatic workers**

| Characteristic | Symptomatic with  positive PCR | Symptomatic with  positive serology | *p*-value |
| --- | --- | --- | --- |
| No. workers | 539 | 197 | - |
| Symptoms |  |  |  |
| Fever (> 37.5ºC) | 165 (30.6) | 54 (27.4) | 0.414 |
| Fever (> 38.0ºC) | 245 (45.6) | 67 (34.0) | **0.005** |
| Myalgia | 397 (73.7) | 133 (67.5) | 0.115 |
| Headache | 414 (76.8) | 134 (68.0) | **0.015** |
| Sore throat | 215 (39.9) | 76 (38.6) | 0.799 |
| Cough | 398 (73.8) | 131 (66.5) | 0.052 |
| Dyspnea | 164 (30.4) | 48 (24.4) | 0.118 |
| Anosmia | 265 (49.2) | 45 (22.8) | **< 0.001** |
| Ageusia | 241 (44.7) | 39 (19.8) | **< 0.001** |
| Disease severity |  |  |  |
| Pneumonia | 64 (11.9) | 24 (12.2) | 0.898 |
| Hospital admission | 33 (6.1) | 1 (0.5) | **< 0.001** |
| ICU admission | 3 (0.6) | 0 (0.0) | 0.568 |
| Outcome (death) | 0 (0.0) | 0 (0.0) | 1.000 |

**Statistics:** Values are expressed as absolute count (percentage). *P*-values were calculated by two-tailed Fisher's exact test. Significant differences are shown in bold. **Abbreviations**: *p*-value: level of significance; ICU: Intensive Care Unit.
