# Supplementary material for: Severe acute respiratory coronavirus virus 2 (SARS-CoV-2) infection among hospital workers in a severely affected institution in Madrid, Spain: A surveillance cross-sectional study
Source: Infect Control Hosp Epidemiol. 2020 Oct 29:1–7. doi: 10.1017/ice.2020.1303 (PMC7691660; doi:10.1017/ice.2020.1303)
Supplement: Supplementary file 1 [file S0899823X20013033sup.zip › S0899823X20013033supp002.docx]

**Supplementary Table 1. Management of COVID-19 patients during the pandemic**

| **Initial measures for IPC in emergency department** |
| --- |
| - Our emergency department created an area to treat patients with respiratory diseases separated from the rest of the pathologies. - This area included a negative pressure room that was available to keep patients with suspected COVID-19 awaiting the results of the PCR. |
| **Employed PPE for patient care** |
| - PPE used for general patient care consisted of surgical mask, medical scrub and disposable gloves. - Recommended PPE for care of COVID-19 patients consisted of disposable medical caps, FFP2 masks, disposable medical protective clothing (waterproof), disposable gloves and protective goggles / protective screens. - When an AGP was necessary, the involved workers wore FFP3 masks. |
| **Measures taken as the pandemic progressed in emergency department** |
| - Due to the increase in the number of COVID-19 cases in our health area, those initial measures were insufficient to assume the outstanding number of patients with suspected COVID-19. In order to overcome this limitation: - The emergency department was then divided into “clean areas” (patients without COVID-19) and “dirty areas” (patients with COVID-19). - Patients with suspected COVID were taken to a “dirty area”, until the result of the PCR. If it was negative, they went to the “clean area”. |
| **Measures taken as the pandemic progressed in hospitalization** |
| - Hospitalization was divided into COVID areas and non-COVID areas. - Within the COVID areas, clean areas (nursing controls and medical offices) and dirty areas (patient rooms) were established, with an intermediate area enabled to put on and take of the PPE. |
| **Problems faced during the pandemic** |
| - The organization was quite similar in the rest of the Spanish hospitals. The problem in Spain, as in other areas of Europe, was the shortage of PPE. - Because of the difficulties in having PPE, we tried to keep the division of the “clean” and “dirty”, in order to assure the availability of one PPE per professional and day. |

**Abbreviations**: IPC: infection prevention control; PPE: personal protective equipment; AGP: aerosol generating procedures.
